# HALP score outperforms systemic inflammatory biomarkers for prognosis in locally advanced rectal cancer

**DOI:** 10.17305/bb.2026.13845

**Published:** 2026-02-03

**Authors:** Peipei Shen, Tiantian Yang, Yawen Cong, Bin Zhang, Yu Xu, Benjie Xu, Shengjun Ji, Yutian Zhao, Yong Mao

**Affiliations:** 1 Department of Radiotherapy and Oncology, Affiliated Hospital of Jiangnan University, Wuxi, Jiangsu, China; 2 Wuxi Medical College, Jiangnan University, Wuxi, Jiangsu, China; 3 Department of Anesthesiology, The Affiliated Hospital of Jiangnan University, Wuxi, Jiangsu, China; 4 Department of Outpatient Chemotherapy, Harbin Medical University Cancer Hospital, Harbin, Heilongjiang, China; 5 Department of Radiotherapy and Oncology, Suzhou Municipal Hospital, The Affiliated Suzhou Hospital of Nanjing Medical University, Gusu School, Nanjing Medical University, Suzhou, Jiangsu, China; 6 Department of Oncology, Affiliated Hospital of Jiangnan University, Wuxi, Jiangsu, China

**Keywords:** Locally advanced rectal cancer, inflammation, biomarker, prognosis

## Abstract

The prognostic value of systemic inflammatory and nutritional biomarkers in patients with locally advanced rectal cancer (LARC) remains inadequately defined. This multicenter retrospective study comprehensively assessed the prognostic performance of twelve inflammation-based indices, aiming to identify the most informative biomarker for patients undergoing neoadjuvant chemoradiotherapy followed by surgery. We analyzed data from 427 patients with stage II–III LARC treated at three medical centers between 2010 and 2021. Twelve biomarkers, derived from routine pretreatment blood parameters—including hemoglobin, albumin, neutrophils, lymphocytes, monocytes, and platelets—were evaluated for their association with overall survival (OS) and disease-free survival (DFS). The prognostic performance was measured using the concordance index (C-index), time-dependent area under the receiver operating characteristic curve (time-AUC), and Brier score. Among the evaluated biomarkers, the hemoglobin–albumin–lymphocyte–platelet (HALP) score exhibited robust and consistent prognostic performance. For OS, HALP achieved a C-index of 0.687 and a time-AUC of 0.668, along with the lowest Brier score (0.134); similar results were observed for DFS (C-index 0.675, time-AUC 0.665). Patients with low HALP scores had significantly worse OS and DFS compared to those with high HALP scores. Multivariate Cox regression analysis confirmed low HALP as an independent risk factor for OS (HR = 3.937, 95% CI: 2.445–6.329; *P* < 0.001) and DFS (HR = 2.212, 95% CI: 1.577–3.096; *P* < 0.001). Nomograms integrating HALP with key clinicopathological variables provided incremental prognostic value, demonstrating good discrimination and calibration at 12, 36, and 60 months. These findings indicate that HALP is a simple and cost-effective biomarker for prognostic stratification in LARC.

## Introduction

Colorectal cancer (CRC) is one of the most commonly diagnosed malignancies worldwide and a leading cause of cancer-related mortality [1]. Rectal cancer constitutes approximately 35%–40% of all CRC cases, with nearly half of patients diagnosed at a locally advanced stage [2, 3]. In China, rectal cancer is more prevalent than colon cancer, with a predominance of middle and low rectal tumors, and a significant proportion of patients presenting with advanced disease at the time of diagnosis [4]. Similar epidemiological trends are observed in Europe, where sociodemographic changes are expected to further elevate incidence rates [2]. Despite the widespread implementation of neoadjuvant chemoradiotherapy (nCRT) followed by total mesorectal excision, clinical outcomes in locally advanced rectal cancer (LARC) remain highly variable [5]. A substantial subset of patients experiences poor treatment response or early recurrence, underscoring the limitations of conventional staging systems in accurately reflecting tumor biology and predicting individual prognosis.

Emerging evidence suggests that systemic inflammation plays a pivotal role in the progression of rectal cancer and its response to treatment. Inflammatory processes influence the tumor microenvironment by modulating immune surveillance, angiogenesis, and metastatic potential [6]. As a result, blood-based inflammatory biomarkers have gained increasing recognition as practical prognostic tools due to their accessibility, low cost, and reproducibility. Numerous inflammation-related indices have been found to correlate with outcomes in LARC, including the pan-immune-inflammation value (PIV) [7], neutrophil-to-lymphocyte ratio (NLR) [8–10], lymphocyte-to-monocyte ratio (LMR) [10], platelet-to-lymphocyte ratio (PLR) [11], systemic immune-inflammation index (SII) [12], systemic inflammation response index (SIRI) [13], neutrophil–albumin ratio (NAR) [14], and the hemoglobin–albumin–lymphocyte–platelet (HALP) score [15, 16]. These markers encompass various aspects of host immunity, nutritional status, and inflammatory burden. However, previous studies have predominantly focused on one or a limited number of indices, leading to heterogeneous and sometimes conflicting results. To date, no study has systematically compared a comprehensive panel of inflammatory markers to determine which provides superior prognostic value in LARC.

In this context, the present study aimed to comprehensively evaluate and compare 12 blood-based inflammatory markers, including PIV, NLR, LMR, PLR, SII, SIRI, NAR, the lymphocyte–albumin ratio (LA), neutrophil–monocyte ratio (NM), neutrophil–platelet ratio (NP), monocyte–platelet ratio (MP), and HALP, in LARC patients treated with nCRT followed by surgery. By examining their associations with clinicopathological characteristics and survival outcomes, we sought to identify the most informative inflammatory indicator and to establish a more reliable and clinically applicable tool for prognostic stratification and individualized management of patients with LARC.

## Materials and methods

### Patients selection

This multicenter retrospective study included consecutive patients with LARC treated between April 2010 and January 2021 at three institutions: Center 1, The Affiliated Hospital of Jiangnan University; Center 2, The Affiliated Suzhou Hospital of Nanjing Medical University; and Center 3, Harbin Medical University Cancer Hospital. LARC was defined as clinical stage II–III disease (cT3–4 and/or cN^+^, M0) based on pelvic magnetic resonance imaging and/or contrast-enhanced computed tomography. Eligible patients had histologically confirmed rectal adenocarcinoma, received standard nCRT followed by curative total mesorectal excision, and had complete baseline clinical and laboratory data obtained prior to neoadjuvant treatment. Patients were excluded if they had distant metastasis, hematologic or autoimmune disorders, received any anticancer therapy before nCRT, underwent non-standard or incomplete neoadjuvant regimens, or had missing clinical, pathological, laboratory, or follow-up information. Following screening, 124 patients from Center 1, 108 from Center 2, and 195 from Center 3 were included, resulting in a total cohort of 427 patients for final analysis (Figure S1).

Tumor staging was assigned according to the 8th edition of the American Joint Committee on Cancer (AJCC) tumor–node–metastasis (TNM) classification. Patients were followed every 3 months during the first 2 years after surgery, every 6 months in years 3–5, and annually thereafter, with evaluations including physical examinations, laboratory testing, tumor marker assessments, and imaging studies as appropriate; structured telephone follow-up was performed when clinic visits were not feasible. Follow-up continued until January 2025. The present study adhered to the Declaration of Helsinki and was approved by the Affiliated Hospital of Jiangnan University, the Affiliated Suzhou Hospital of Nanjing Medical University, and the Harbin Medical University Cancer Hospital.

### Treatment protocol

Neoadjuvant chemoradiotherapy was administered according to institutional standards consistent with contemporary clinical guidelines for LARC. Radiotherapy was delivered using a long-course schedule, with a total dose of 45.0–50.4 Gy in 25–28 fractions, once daily, five days per week. Concurrent chemotherapy primarily involved fluoropyrimidine-based regimens, including oral capecitabine (825 mg/m^2^ twice daily on radiotherapy days) or continuous-infusion 5-fluorouracil, based on institutional preference. Surgery with total mesorectal excision was typically performed 6–10 weeks after completing nCRT. Postoperative adjuvant chemotherapy was recommended for patients with high-risk pathological features, such as ypT3–4 and/or positive lymph nodes, although specific regimens and treatment decisions varied by center and over time. All treatments were delivered as part of routine clinical practice.

### Data collection and definition of variables

This study collected a comprehensive set of demographic, clinicopathological, and laboratory variables. Baseline patient characteristics included age and gender. Tumor-related variables comprised histological differentiation, pretreatment serum carcinoembryonic antigen (CEA) levels, and clinical tumor stage determined according to the 8th edition of the AJCC TNM classification. Pathological response after nCRT was assessed using postoperative pathological staging, including ypT stage and ypN status.

Pre-treatment hematological parameters were obtained from peripheral blood samples collected prior to neoadjuvant therapy and included absolute counts of neutrophils, lymphocytes, monocytes, and platelets, as well as hemoglobin and serum albumin levels. Based on these parameters, 12 systemic inflammatory and nutritional biomarkers were calculated: NLR, LMR, PLR, SII, SIRI, NAR, LA, NM, NP, MP, PIV, and HALP. The detailed calculation formulas are provided in Table S1. The selection of these indices was intended to facilitate a comprehensive comparison of widely used inflammatory markers and emerging composite indicators in patients with LARC. Overall survival (OS) was defined as the interval from the date of radical surgery to death from any cause or the last follow-up. Disease-free survival (DFS) was calculated from the date of surgery to the first documented recurrence or the most recent follow-up for patients without recurrence.

### Evaluation metrics for inflammatory biomarkers

A comprehensive assessment of the prognostic performance of 12 systemic inflammatory biomarkers was conducted using several complementary metrics. Discrimination was evaluated with Harrell’s concordance index (C-index) and time-dependent receiver operating characteristic curves, quantified by the area under the curve (time-AUC). Higher values of these metrics indicate superior predictive accuracy. Model calibration was assessed using the time-dependent Brier score, which ranges from 0–1, where lower values signify better agreement between predicted and observed outcomes.

Time-dependent receiver operating characteristic (ROC) curves and time-AUC were estimated utilizing inverse probability of censoring weighting methods to adequately account for right-censored survival data. The time-dependent Brier score was calculated at 12, 36, and 60 months to evaluate model calibration. The C-index was employed to assess overall discriminative ability, with confidence intervals for the C-index, time-AUC, and Brier score obtained through bootstrap resampling with 500 iterations.

### Development and assessment of nomogram

Independent prognostic factors for OS and DFS were initially screened using univariate Cox proportional hazards analyses and subsequently identified through multivariate stepwise Cox regression. A baseline clinical model was established using independent predictors of OS and DFS, excluding HALP. Nomogram models were then constructed by integrating HALP with the remaining independent prognostic variables to provide individualized survival estimates. Model performance was evaluated using time-dependent ROC curves and calibration plots. The incremental predictive value of the nomogram compared to the clinical model and TNM staging system was quantified using net reclassification improvement (NRI) and integrated discrimination improvement (IDI), with confidence intervals derived from bootstrap resampling.

Internal validation and optimism correction were performed through bootstrap resampling (500 iterations). In each bootstrap sample, the model—including the same variable-selection procedure—was refit, and performance (C-index, time-dependent AUC, Brier score, and calibration) was evaluated in both the bootstrap sample and the original cohort. Optimism was estimated as the mean difference between apparent and test performance across resamples, and optimism-corrected performance was obtained by subtracting this optimism from the apparent estimates.

### Units and computation

Hemoglobin was recorded in g/L, serum albumin in g/L, and absolute neutrophil, lymphocyte, monocyte, and platelet counts in ×10^9^/L at all participating centers. Prior to calculating biomarkers, raw laboratory values were harmonized to these units. When hemoglobin was reported in g/dL, values were converted to g/L by multiplying by 10; similarly, when albumin was reported in g/dL, values were also converted by multiplying by 10. Cell counts reported as ×10^3^/µL were directly equivalent to ×10^9^/L and were therefore utilized without conversion. All indices were subsequently calculated from the harmonized values according to the formulas listed in Table S1. No additional scaling, normalization, or center-specific recalibration was performed.

### Assessment of proportional hazards, nonlinearity, and center heterogeneity

The proportional hazards assumption for HALP in both OS and DFS models was evaluated using Schoenfeld residuals. To investigate whether the association between HALP and survival outcomes deviated from linearity, restricted cubic spline functions with three knots were fitted into the Cox proportional hazards models. Wald χ^2^ tests were employed to assess the overall linear and nonlinear components of HALP. To explore potential center-level heterogeneity within this multicenter cohort, univariate Cox regression analyses including center as a categorical variable were conducted for OS and DFS.

### Statistical analysis

The normality of continuous variables was assessed using the Kolmogorov–Smirnov test. Data were expressed as mean ± standard deviation (SD) for normally distributed variables or as median with interquartile range (IQR) for non-normally distributed variables. Comparisons between groups were performed using the Student’s *t*-test or the Mann–Whitney *U* test for continuous variables, and the chi-square test or Fisher’s exact test for categorical variables, as appropriate. Potential prognostic factors were initially examined through univariate Cox proportional hazards analysis, and variables with a *P* value < 0.05 were subsequently entered into a multivariate Cox regression model using an inverse stepwise selection method. All statistical analyses were conducted using R software (version 4.2.1), with a two-sided *P* value < 0.05 considered statistically significant.

### Ethical statement

The studies involving human participants were reviewed and approved by the Ethics Committees of The Affiliated Hospital of Jiangnan University, The Affiliated Suzhou Hospital of Nanjing Medical University, and Harbin Medical University Cancer Hospital. Written informed consent was obtained from all patients/participants to participate in this study.

## Results

### Patient characteristics

A total of 427 patients with LARC were included in the analysis. More than half of the cohort (55.27%) was younger than 60 years, while 44.73% were aged 60 years or older. Male patients predominated, accounting for 65.34% of cases, whereas females represented 34.66%. Most tumors were moderately differentiated (79.86%), with well-differentiated and poorly differentiated tumors observed in 9.60% and 10.54% of patients, respectively. At diagnosis, 42.39% of patients had elevated pretreatment serum CEA levels (≥5 ng/mL). According to the 8th edition of the AJCC clinical TNM staging system, 32.79% of patients were classified as stage II, and 67.21% as stage III. Postoperative pathological assessment following nCRT indicated that 17.33% of patients achieved ypT0 status, 23.19% were staged as ypT1–2, and the majority (59.48%) remained ypT3–4. Lymph node metastasis was absent in 73.54% of patients, while 26.46% exhibited positive ypN status. Baseline systemic inflammatory and nutritional biomarkers demonstrated considerable variability across the cohort. The median (IQR) values were 2.40 (1.77–3.33) for NLR, 3.29 (2.19–4.04) for LMR, 143.75 (108.06–182.86) for PLR, 538.96 (379.32–811.14) for SII, and 1.30 (0.82–1.94) for SIRI. The median NAR was 1.12 (0.85–1.54), while composite indices showed median values of 6.21 (4.85–7.80) for LA, 2.31 (1.46–3.74) for NM, 1031.58 (668.87–1458.08) for NP, 132.21 (84.93–184.62) for MP, 322.64 (191.04–478.84) for PIV, and 23.43 (11.56–37.47) for HALP. Collectively, these data summarize the demographic, clinicopathological, and inflammatory characteristics of the study population (Table 1). The median follow-up duration was 47 months (IQR, 28–83), ranging from 2–158 months. During the follow-up period, 101 deaths were recorded for OS, and 156 events were noted for DFS.

**Table 1:** Baseline clinicopathological characteristics of patients with locally advanced rectal cancer in this study

**Variables**	**All patients (*n* ═ 427)**
Age (year, %)	
< 60	236 (55.27)
≥ 60	191 (44.73)
Gender (%)	
Female	148 (34.66)
Male	279 (65.34)
Differentiation (%)	
Well	41 (9.60)
Moderate	341 (79.86)
Poor	45 (10.54)
CEA pretreatment (%)	
≥ 5 ng/m	181 (42.39)
< 5 ng/mL	246 (57.61)
cTNM (AJCC 8th edition, %)	
II	140 (32.79)
III	287 (67.21)
ypT (%)	
ypT0	74 (17.33)
ypT1-2	99 (23.19)
ypT3-4	254 (59.48)
ypN (%)	
Negative	314 (73.54)
Positive	113 (26.46)
NLR (median [IQR])	2.40 (1.77, 3.33)
LMR (median [IQR])	3.29 (2.19, 4.04)
PLR (median [IQR])	143.75 (108.06, 182.86)
SII (median [IQR])	538.96 (379.32, 811.14)
SIRI (median [IQR])	1.30 (0.82, 1.94)
NAR (median [IQR])	1.12 (0.85, 1.54)
LA (median [IQR])	6.21 (4.85, 7.80)
NM (median [IQR])	2.31 (1.46, 3.74)
NP (median [IQR])	1031.58 (668.87, 1458.08)
MP (median [IQR])	132.21 (84.93, 184.62)
PIV (median [IQR])	322.64 (191.04, 478.84)
HALP (median [IQR])	23.43 (11.56, 37.47)

Abbreviations: CEA: Carcinoembryonic antigen; HALP: Hemoglobin, albumin, lymphocyte, and platelet score; IQR: Interquartile range; LA: Lymphocyte × albumin; LMR: Lymphocyte-to-monocyte ratio; MP: Monocyte ×. platelet; NAR: Neutrophil-to-albumin ratio; NLR: Neutrophil-to-lymphocyte ratio; NM: Neutrophil × monocyte; NP: Neutrophil × platelet; PIV: Pan-immune-inflammation value; PLR: Platelet-to-lymphocyte ratio; SII: Systemic immune-inflammation index; SIRI: Systemic inflammation response index.

### Optimal survival prediction inflammation biomarkers

**Figure 1.**
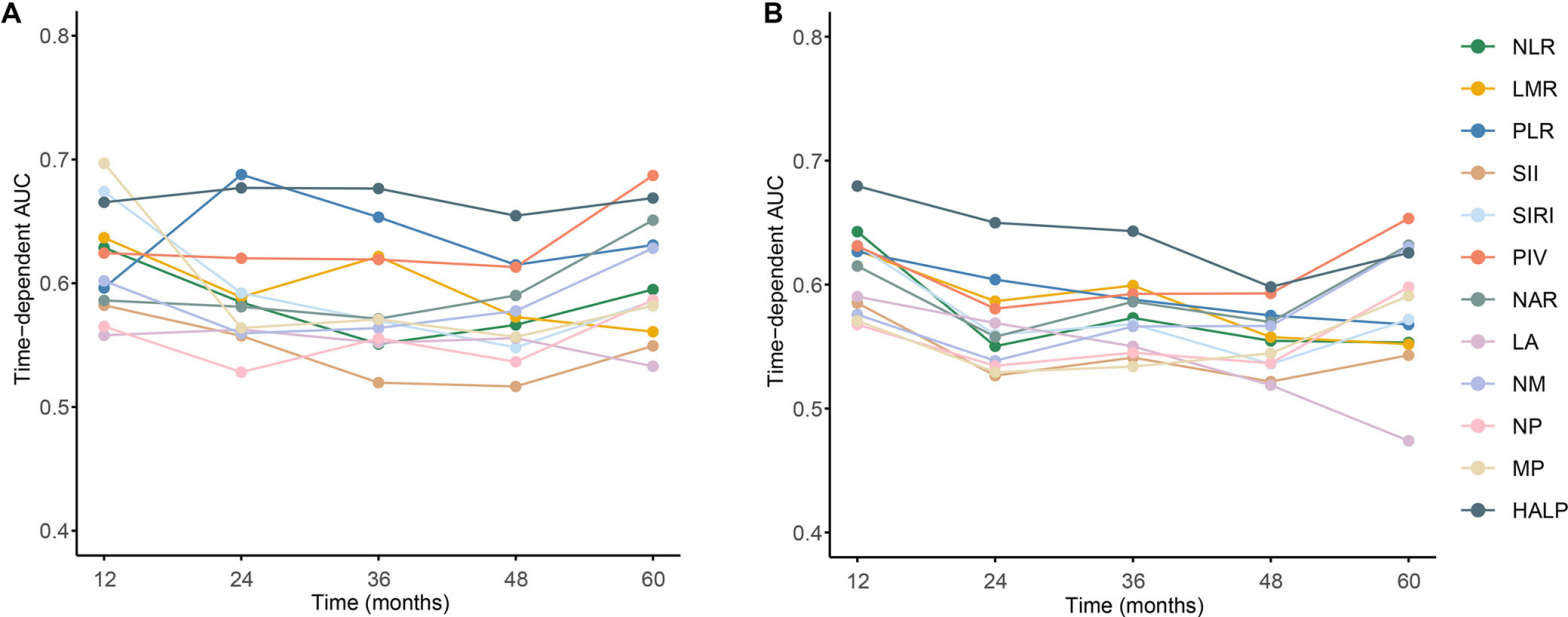
**Time-dependent discriminatory performance of systemic inflammation–based biomarkers in locally advanced rectal cancer.** (A) Time-dependent AUC for overall survival at 12, 24, 36, 48, and 60 months of follow-up for twelve inflammation-related indices (NLR, LMR, PLR, SII, SIRI, PIV, NAR, LA, NM, NP, MP, and HALP). (B) Time-dependent AUC for disease-free survival at the same follow-up time points for the same set of indices. Higher AUC values indicate better prognostic discrimination; HALP demonstrates the highest and most stable AUC across time points for both endpoints. Abbreviations: AUC: Area under the receiver operating characteristic curve; DFS: Disease-free survival; HALP: Hemoglobin–albumin–lymphocyte–platelet score; LA: Lymphocyte–albumin ratio; LARC: Locally advanced rectal cancer; LMR: Lymphocyte-to-monocyte ratio; MP: Monocyte–platelet ratio; NAR: Neutrophil–albumin ratio; NLR: Neutrophil-to-lymphocyte ratio; NM: Neutrophil–monocyte ratio; NP: Neutrophil–platelet ratio; OS: Overall survival; PIV: Pan-immune-inflammation value; PLR: Platelet-to-lymphocyte ratio; ROC: Receiver operating characteristic; SII: Systemic immune-inflammation index; SIRI: Systemic inflammation response index.

As illustrated in Figure 1 and summarized in Table 2, the prognostic value of 12 systemic inflammatory biomarkers for OS and DFS was comprehensively evaluated. Among all indices, HALP exhibited the most favorable and stable predictive performance. For OS, HALP achieved the highest C-index (0.687, 95% CI: 0.612–0.767), the highest time-dependent AUC (0.668, 95% CI: 0.603–0.734), and the lowest time-Brier score (0.134, 95% CI: 0.120–0.151). A similar trend was observed for DFS, with HALP yielding a C-index of 0.675 (95% CI: 0.607–0.743), a time-AUC of 0.665 (95% CI: 0.605–0.724), and the lowest time-Brier score (0.192, 95% CI: 0.176–0.205).

In contrast, commonly used inflammatory markers such as NLR, PLR, SII, and NAR demonstrated only moderate prognostic discrimination, while composite indices, including PIV and SIRI, exhibited intermediate performance. NP and MP showed relatively limited predictive ability, particularly for DFS. Time-dependent AUC curves further confirmed that HALP consistently outperformed the other biomarkers across multiple follow-up time points for both OS (Figure 1A) and DFS (Figure 1B), indicating its superior discriminatory capacity and overall prognostic robustness in patients with LARC.

### Characteristics and survival analysis of HALP

Patients were stratified into low HALP (< 23.43) and high HALP (≥ 23.43) groups according to the median HALP value (Table 3). No significant differences were observed between the two groups concerning age (*P* ═ 0.112), gender (*P* ═ 0.731), tumor differentiation (*P* ═ 0.812), pretreatment CEA level (*P* ═ 0.091), clinical TNM stage (*P* ═ 0.454), ypT stage (*P* ═ 0.501), or ypN status (*P* ═ 0.491). In contrast, significant differences were identified in multiple inflammation-related indices. Compared with patients in the high HALP group, those with low HALP exhibited significantly higher levels of NLR, PLR, SII, SIRI, PIV, NAR, NM, NP, and MP, along with significantly lower LMR and LA (all *P* < 0.05).

**Table 2:** Comparative analysis of twelve inflammatory biomarkers in predicting overall survival and disease-free survival

**Biomarkers**	**Overall survival**			**Disease-free survival**		
	**C-index (95% CI)**	**Time-AUC (95% CI)**	**Time-Brier score (95% CI)**	**C-index** **(95% CI)**	**Time-AUC (95% CI)**	**Time-Brier score (95% CI)**
PIV	0.609 (0.548, 0.679)	0.585 (0.492, 0.678)	0.139 (0.121, 0.156)	0.591 (0.552, 0.640)	0.603 (0.519, 0.676)	0.197 (0.180, 0.210)
NLR	0.577 (0.518, 0.636)	0.554 (0.470, 0.648)	0.137 (0.119, 0.151)	0.557 (0.519, 0.606)	0.568 (0.494, 0.642)	0.196 (0.179, 0.208)
LMR	0.629 (0.571, 0.687)	0.637 (0.542, 0.731)	0.136 (0.119, 0.151)	0.565 (0.516, 0.613)	0.570 (0.496, 0.644)	0.197 (0.181, 0.208)
PLR	0.615 (0.556, 0.675)	0.605 (0.510, 0.700)	0.139 (0.119, 0.157)	0.521 (0.471, 0.571)	0.521 (0.446, 0.597)	0.197 (0.182, 0.210)
SII	0.573 (0.515, 0.631)	0.594 (0.507, 0.681)	0.139 (0.121, 0.156)	0.545 (0.497, 0.594)	0.551 (0.478, 0.625)	0.197 (0.181, 0.210)
SIRI	0.616 (0.564, 0.668)	0.603 (0.520, 0.686)	0.136 (0.120, 0.152)	0.570 (0.523, 0.618)	0.588 (0.516, 0.660)	0.194 (0.177, 0.206)
NAR	0.587 (0.528, 0.646)	0.596 (0.501, 0.691)	0.138 (0.119, 0.158)	0.558 (0.508, 0.607)	0.570 (0.496, 0.644)	0.197 (0.179, 0.210)
LA	0.546 (0.489, 0.602)	0.548 (0.457, 0.638)	0.138 (0.119, 0.155)	0.520 (0.474, 0.576)	0.532 (0.460, 0.613)	0.198 (0.181, 0.209)
NM	0.575 (0.518, 0.631)	0.586 (0.495, 0.678)	0.139 (0.117, 0.155)	0.541 (0.494, 0.589)	0.553 (0.480, 0.626)	0.197 (0.180, 0.210)
NP	0.538 (0.479, 0.597)	0.554 (0.458, 0.651)	0.139 (0.121, 0.156)	0.518 (0.469, 0.568)	0.534 (0.459, 0.609)	0.197 (0.181, 0.209)
MP	0.563 (0.508, 0.617)	0.594 (0.510, 0.678)	0.139 (0.123, 0.153)	0.517 (0.469, 0.566)	0.532 (0.457, 0.606)	0.197 (0.179, 0.209)
HALP	0.687 (0.612, 0.767)	0.668 (0.603, 0.734)	0.134 (0.120, 0.151)	0.675 (0.607, 0.743)	0.665 (0.605, 0.724)	0.192 (0.176, 0.205)

Abbreviations: C-index: Concordance index; CI: Confidence interval; LA: Lymphocyte × albumin; LMR: Lymphocyte-to-monocyte ratio; MP: Monocyte × platelet; NAR: Neutrophil-to-albumin ratio; NLR: Neutrophil-to-lymphocyte ratio; NM: Neutrophil × monocyte; NP: Neutrophil × platelet; PIV: Pan-immune-inflammation value; PLR: Platelet-to-lymphocyte ratio; SII: Systemic immune-inflammation index; SIRI: Systemic inflammation response index; Time-AUC: Time-dependent area under the receiver operating characteristic curve; Time-Brier Score: Time-dependent Brier score.

**Table 3:** Association of the HALP with clinicopathological characteristics in patients with locally advanced rectal cancer

**Variables**	**Low HALP (*n* ═ 214)**	**High HALP (*n* ═ 213)**	***P* value**
Age (year, %)			0.112
< 60	127 (59.35)	109(51.17)	
≥ 60	87 (40.65)	104 (48.83)	
Gender (%)			0.731
Female	72 (33.64)	76 (35.68)	
Male	142 (66.36)	137 (64.32)	
Differentiation (%)			0.812
Well	22 (10.28)	19 (8.92)	
Moderate	171 (79.91)	170 (79.81)	
Poor	21 (9.81)	24 (11.27)	
CEA pretreatment (%)			0.091
≥ 5 ng/m	100 (46.73)	81 (38.03)	
< 5 ng/mL	114 (53.27)	132 (61.97)	
cTNM (AJCC 8th edition, %)			0.454
II	66 (30.84)	74 (34.74)	
III	148 (69.16)	139 (65.26)	
ypT (%)			0.501
ypT0	33 (15.42)	41 (19.25)	
ypT1-2	53 (24.77)	46 (21.60)	
ypT3-4	128 (59.81)	126 (59.15)	
ypN (%)			0.491
Negative	161 (75.23)	153 (71.83)	
Positive	53 (24.77)	60 (28.17)	
NLR (median [IQR])	2.75 (2.01, 3.62)	2.12 (1.65, 2.93)	<0.001
LMR (median [IQR])	2.71 (2.01, 3.47)	3.73 (2.78, 5.13)	<0.001
PLR (median [IQR])	153.25 (116.88, 186.77)	134.53 (100.00, 174.67)	0.007
SII (median [IQR])	610.72 (439.42, 929.20)	498.69 (324.95, 699.32)	<0.001
SIRI (median [IQR])	1.62 (1.17, 2.32)	1.01 (0.63, 1.53)	<0.001
PIV (median [IQR])	374.03 (257.77, 603.59)	245.22 (143.91, 367.06)	<0.001
NAR (median [IQR])	1.23 (1.03, 1.63)	0.99 (0.77, 1.33)	<0.001
LA (median [IQR])	5.53 (4.54, 6.72)	6.93 (5.18, 9.06)	<0.001
NM (median [IQR])	2.63 (1.80, 3.90)	1.81 (1.19, 3.25)	<0.001
NP (median [IQR])	1163.41 (712.05, 1557.50)	936.96 (620.60, 1326.51)	0.002
MP (median [IQR])	149.88 (104.15, 197.18)	116.85 (78.80, 157.38)	<0.001

Abbreviations: CEA: Carcinoembryonic antigen; HALP: Hemoglobin, albumin, lymphocyte, and platelet score; IQR: Interquartile range; LA: Lymphocyte × albumin; LMR: Lymphocyte-to-monocyte ratio; MP: Monocyte × platelet; NAR: Neutrophil-to-albumin ratio; NLR: Neutrophil-to-lymphocyte ratio; NM: Neutrophil × monocyte; NP: Neutrophil × platelet; PIV: Pan-immune-inflammation value; PLR: Platelet-to-lymphocyte ratio; SII: Systemic immune-inflammation index; SIRI: Systemic inflammation response index.

**Table 4:** Univariate and multivariate analyses of prognostic factors in locally advanced rectal cancer

**Variables**	**Overall survival**				**Disease free survival**			
	**Univariate analysis**		**Multivariate analysis**		**Univariate analysis**		**Multivariate analysis**	
	**HR (95%CI)**	***P* value**	**HR (95%CI)**	***P* value**	**HR (95%CI)**	***P* value**	**HR (95%CI)**	***P* value**
HALP (Low vs. High)	3.774 (2.358--6.061)	<0.001	3.937 (2.445--6.329)	<0.001	2.347 (1.678--3.279)	<0.001	2.212 (1.577--3.096)	<0.001
Age (≥ 60 year vs. < 60 year)	1.331 (0.897--1.962)	0.156			1.062 (0.774--1.463)	0.702		
Gender (male vs. Female)	0.919 (0.611--1.381)	0.684			0.967 (0.694--1.354)	0.841		
Differentiation (poor vs. Moderate)	1.564 (0.867--2.812)	0.137			1.152 (0.695--1.926)	0.578		
Differentiation (well vs. Moderate)	0.675 (0.309--1.454)	0.309			0.908 (0.523--1.584)	0.733		
CEA (< 5 ng/ml vs. ≥ 5 ng/mL)	0.792 (0.536--1.172)	0.242			0.585 (0.426--0.804)	0.001	0.669 (0.485--0.922)	0.014
TNM (III vs. II)	1.072 (0.711--1.614)	0.744			1.081 (0.772--1.502)	0.665		
ypT (ypT1-2 vs. ypT0)	2.31 (0.993--5.385)	0.052	1.752 (0.746--4.124)	0.197	1.843 (0.995--3.422)	0.051	1.682 (0.905--3.124)	0.101
ypT (ypT3-4 vs. ypT0)	2.87 (1.322--6.245)	0.007	2.313 (1.065--5.052)	0.035	2.292 (1.313--4.013)	0.003	2.052 (1.175--3.592)	0.012
ypN (positive vs. Negative)	1.991 (1.316--3.018)	0.001	2.133 (1.403--3.255)	<0.001	1.101 (0.764--1.605)	0.598		

Abbreviations: CEA: Carcinoembryonic antigen; CI: Confidence interval; HALP: Hemoglobin, albumin, lymphocyte, and platelet score; HR: Hazard ratio; TNM: Tumor-node-metastasis.

**Figure 2.**
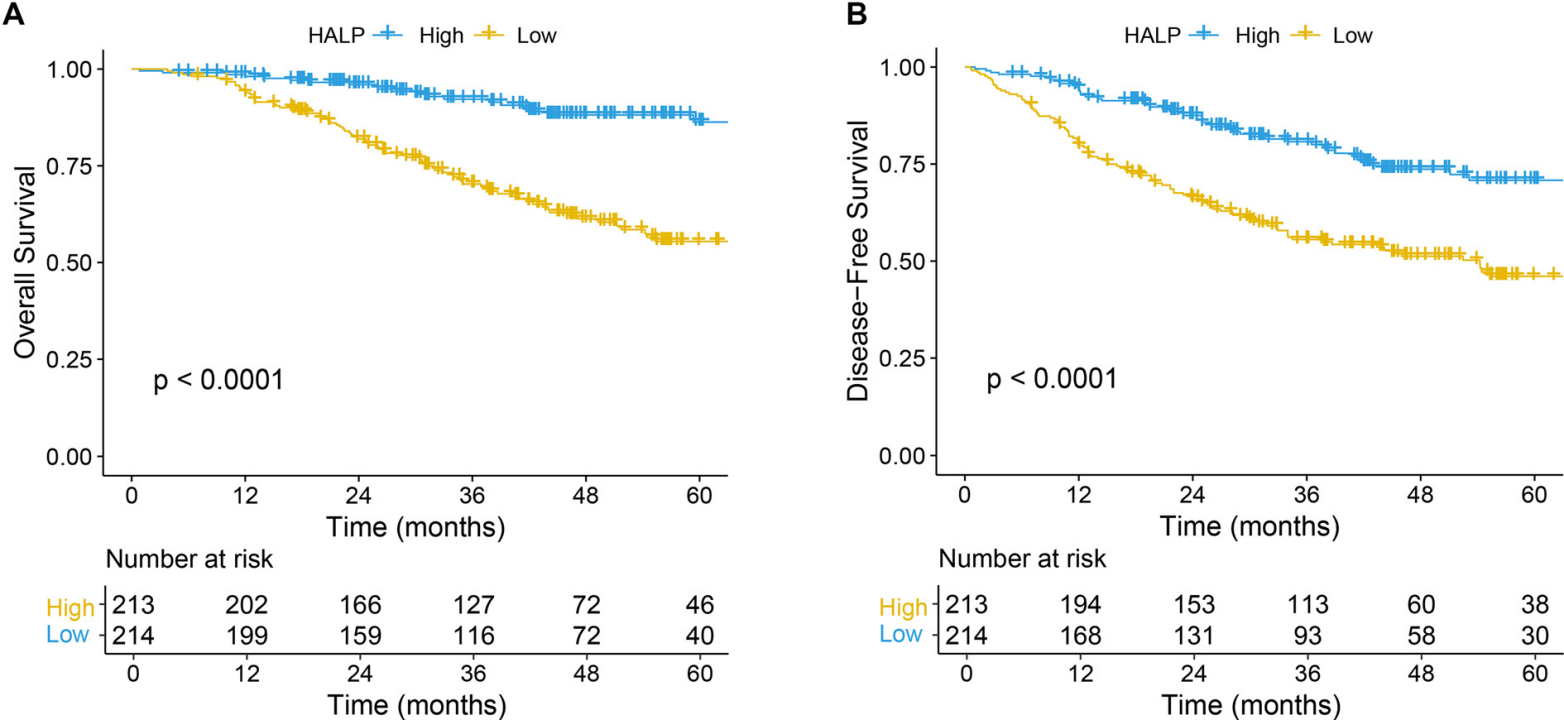
**Kaplan–Meier survival curves stratified by HALP in patients with locally advanced rectal cancer.** (A) Overall survival according to HALP group (high, *n* ═ 213; low, *n* ═ 214); patients with low HALP show significantly poorer survival than those with high HALP (log-rank *P* < 0.0001). (B) Disease-free survival according to HALP group (high, *n* ═ 213; low, *n* ═ 214); low HALP is associated with significantly worse disease-free survival (log-rank *P* < 0.0001). Time is shown in months; tick marks indicate censored observations, and numbers at risk are provided below each panel. Abbreviations: DFS: Disease-free survival; HALP: Hemoglobin–albumin–lymphocyte–platelet score; LARC: Locally advanced rectal cancer; OS: Overall survival.

Kaplan–Meier survival analyses demonstrated that patients with low HALP experienced significantly worse OS and DFS compared to those with high HALP (both *P* < 0.0001; Figure 2A and 2B). To further elucidate the prognostic significance of HALP, univariate and multivariate Cox proportional hazards analyses were performed (Table 4). In univariate analysis, low HALP was strongly associated with inferior OS (HR = 3.774, 95% CI: 2.358--6.061, *P* < 0.001) and DFS (HR = 2.347, 95% CI: 1.678--3.279, *P* < 0.001). After adjustment for potential confounders, HALP remained an independent predictor of both outcomes. In multivariate analysis, low HALP was independently associated with poor OS (HR = 3.937, 95% CI: 2.445--6.329, *P* < 0.001) and DFS (HR = 2.212, 95% CI: 1.577--3.096, *P* < 0.001). Additionally, postoperative pathological tumor stage demonstrated significant prognostic relevance; patients with ypT3–4 disease had a significantly higher risk of death and recurrence compared to those achieving ypT0, both for OS (HR = 2.313, 95% CI: 1.065–5.052, *P* ═ 0.035) and DFS (HR = 2.052, 95% CI: 1.175–3.592, *P* ═ 0.012). Positive ypN status independently predicted worse OS (HR = 2.133, 95% CI: 1.403–3.255, *P* < 0.001), although its association with DFS did not reach statistical significance. Furthermore, elevated pretreatment CEA levels (≥5 ng/mL) were independently associated with reduced DFS (HR = 0.669, 95% CI: 0.485–0.922, *P* ═ 0.014), but not OS in the multivariate analysis. Other variables, including age, sex, tumor differentiation, and clinical TNM stage, were not independently associated with survival outcomes.

### Assessment of proportional hazards, nonlinearity, and center heterogeneity

The proportional hazards assumption for HALP was not violated in either the OS or DFS models, as evidenced by Schoenfeld residual analyses showing no systematic time-dependent trends (Figure S2). In restricted cubic spline analyses treating HALP as a continuous variable, the overall association between HALP and survival outcomes was statistically significant, while tests for nonlinearity were not significant for either OS or DFS, indicating that the effect of HALP on prognosis could be adequately modeled as approximately linear on the log-hazard scale (Figure S3). Additionally, univariate analyses incorporating treatment center demonstrated no significant differences in baseline hazard for OS or DFS among the three participating centers, suggesting minimal center-level heterogeneity in survival outcomes (Table S2).

### Development and assessment of nomograms

**Figure 3.**
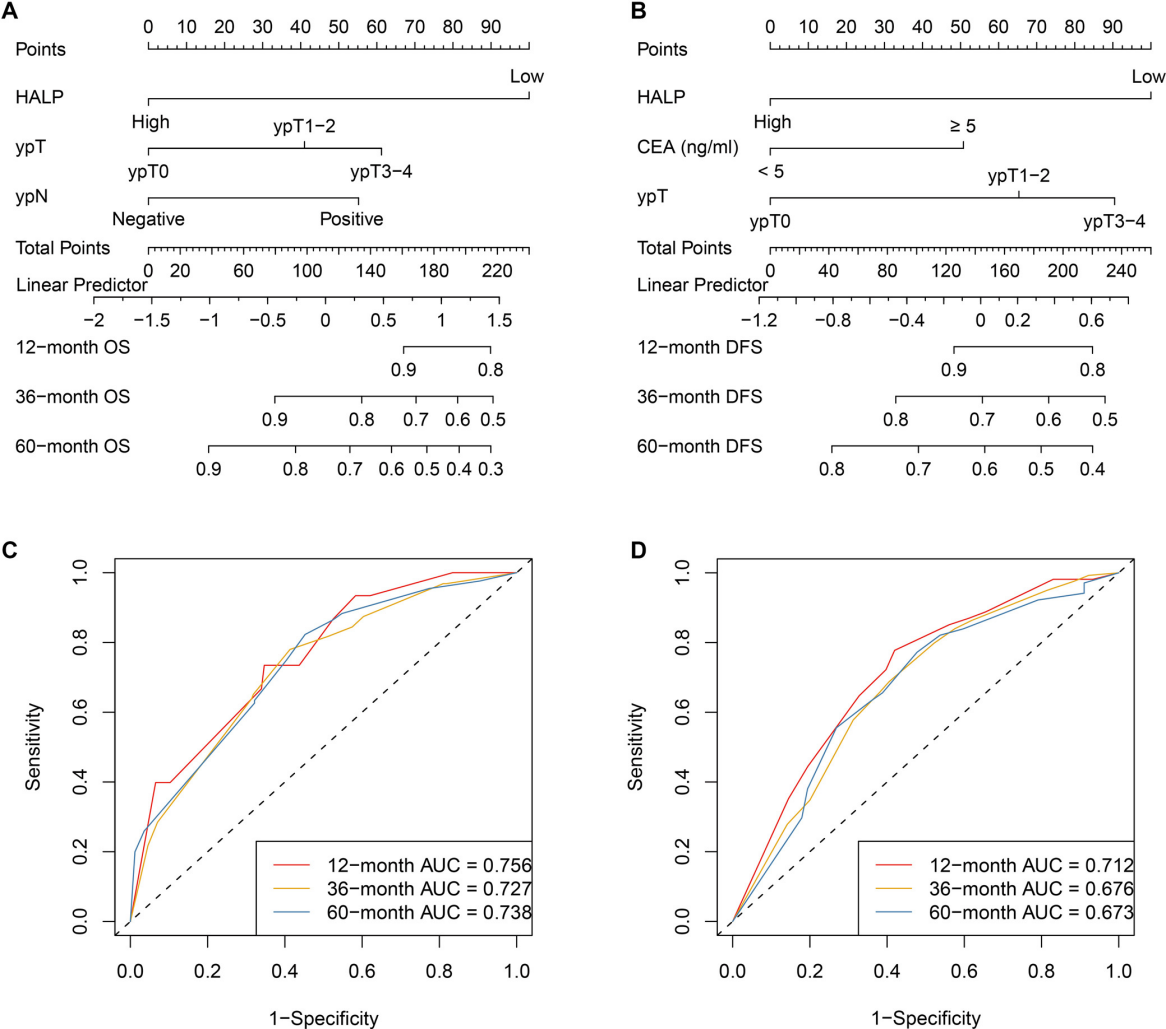
**Nomogram development and discrimination performance for individualized prediction of overall and disease-free survival in locally advanced rectal cancer.** (A) OS nomogram constructed from multivariable Cox regression, incorporating HALP, ypT, and ypN. (B) DFS nomogram incorporating HALP, pretreatment carcinoembryonic antigen (CEA; <5 vs ≥5 ng/ml), and ypT stage. For each nomogram, points assigned to each predictor are summed to obtain total points, which correspond to estimated 12-, 36-, and 60-month survival probabilities. (C) Time-dependent ROC curves evaluating discrimination of the OS nomogram at 12, 36, and 60 months (AUC = 0.756, 0.727, and 0.738, respectively). (D) Time-dependent ROC curves for the DFS nomogram at 12, 36, and 60 months (AUC = 0.712, 0.676, and 0.673, respectively). Abbreviations: AUC: Area under the receiver operating characteristic curve; CEA: Carcinoembryonic antigen; DFS: Disease-free survival; HALP: Hemoglobin–albumin–lymphocyte–platelet score; LARC: Locally advanced rectal cancer; OS: Overall survival; ROC: Receiver operating characteristic; ypN: Postoperative pathological nodal status; ypT: Postoperative pathological tumor stage.

**Figure 4.**
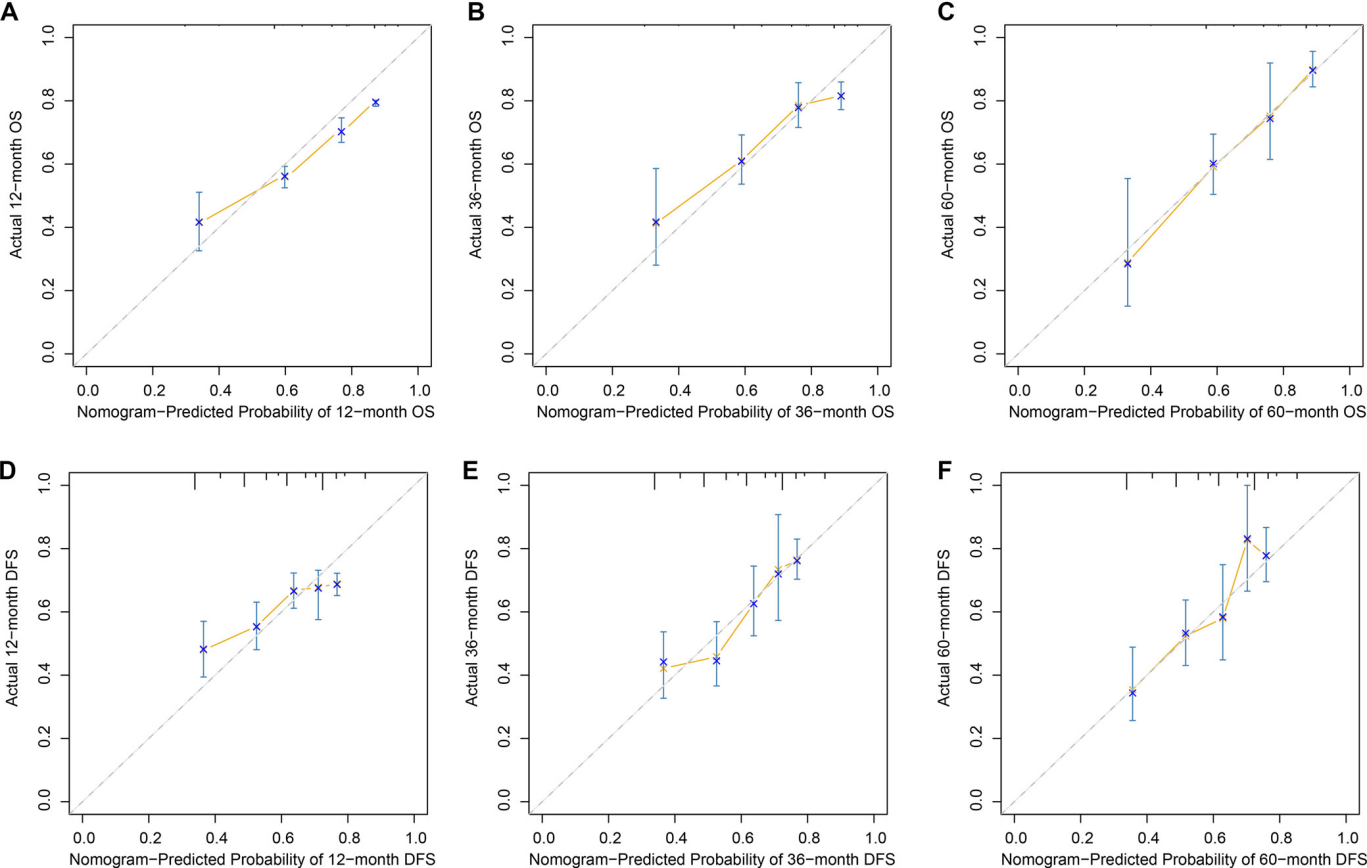
**Calibration of the HALP-based nomograms for overall and disease-free survival in locally advanced rectal cancer.** (A–C) Calibration plots for the OS nomogram at 12 months (A), 36 months (B), and 60 months (C), comparing nomogram-predicted survival probabilities (x-axis) with the observed survival probabilities (y-axis). (D–F) Corresponding calibration plots for the DFS nomogram at 12 months (D), 36 months (E), and 60 months (F). The diagonal reference line indicates perfect agreement; closer alignment of the calibration curve with this line reflects better calibration. Error bars denote uncertainty around the observed estimates. Abbreviations: DFS: Disease-free survival; HALP: Hemoglobin–albumin–lymphocyte–platelet score; LARC: Locally advanced rectal cancer; OS: Overall survival.

Based on the results of multivariate Cox regression analysis, HALP, along with key clinicopathological variables, was identified as an independent prognostic factor for survival outcomes and subsequently incorporated into prognostic model construction. For OS, HALP, ypT, and ypN were integrated to develop the OS nomogram (Figure 3A). For DFS, HALP, pretreatment CEA level, and ypT stage were included in the DFS nomogram (Figure 3B). These nomograms facilitate individualized estimation of survival probability by assigning weighted points to each prognostic factor. The predictive performance of the nomogram models was further evaluated using time-dependent ROC curves. The OS nomogram demonstrated good discriminative ability, with AUC values of 0.756, 0.727, and 0.738 for predicting 12-, 36-, and 60-month OS, respectively (Figure 3C). Similarly, the DFS nomogram exhibited favorable predictive accuracy, achieving AUC values of 0.712, 0.676, and 0.673 at 12, 36, and 60 months, respectively (Figure 3D). Collectively, these findings indicate that nomogram models incorporating HALP provide reliable and clinically meaningful prognostic estimates for patients with LARC. The calibration curves demonstrated a high level of concordance between predicted and actual survival probabilities at 12, 36, and 60 months, supporting the strong calibration and reliable predictive performance of the models for postoperative outcomes (Figure 4 A-F ).

### Model comparison

To compare the predictive performance of different prognostic approaches, the nomogram, TNM staging system, and clinical model were evaluated using the C-index, IDI, and NRI (Table 5). For OS, the nomogram exhibited the best discriminatory ability, with a C-index of 0.705 (95% CI: 0.661–0.749), surpassing that of the TNM system (0.646) and the clinical model (0.682). Relative to the clinical model, the nomogram provided a significant improvement in discrimination, as indicated by an IDI of 0.072 (95% CI: 0.011–0.129, *P* ═ 0.003), and a substantial enhancement in risk reclassification, with an NRI of 0.209 (95% CI: 0.127–0.289, *P* < 0.001). In contrast, the TNM system demonstrated inferior performance, revealing significantly reduced IDI (--0.052, *P* < 0.001) and NRI (--0.162, *P* < 0.001) compared to the clinical model. A similar pattern was observed for DFS, where the nomogram achieved the highest C-index of 0.693 (95% CI: 0.628–0.745), followed by the clinical model (0.662) and the TNM system (0.616). Compared with the clinical model, the nomogram yielded significant gains in predictive accuracy and reclassification, with an IDI of 0.081 (*P* ═ 0.002) and an NRI of 0.228 (*P* < 0.001). Collectively, these findings suggest that the nomogram offers superior prognostic discrimination and risk stratification for both OS and DFS compared to the TNM staging system and the clinical model alone.

## Discussion

In this comprehensive multicenter study, we systematically evaluated 12 systemic inflammatory biomarkers in patients with LARC and found that the HALP score exhibited the strongest and most consistent prognostic value for both OS and DFS. By integrating hemoglobin, albumin, lymphocyte, and platelet levels, HALP encapsulates the intricate interplay between nutritional status, systemic inflammation, and host immune competence. Our findings demonstrate that a low HALP score correlates closely with an unfavorable inflammatory profile and significantly poorer clinical outcomes, independent of established clinicopathological factors. Furthermore, the incorporation of HALP into prognostic nomograms substantially enhanced predictive accuracy and risk stratification compared to the TNM staging system alone, underscoring its potential clinical utility for personalized prognosis assessment in LARC.

The superior prognostic performance of the HALP score observed in our cohort of patients with LARC undergoing nCRT is biologically plausible and aligns with a growing body of literature across various solid tumor types. Unlike single-component inflammatory indices such as NLR, PLR, or SII, HALP integrates four routinely available hematological parameters—hemoglobin, albumin, lymphocyte count, and platelet count—thereby providing a more comprehensive reflection of the host’s immunological, inflammatory, and nutritional status [17–21]. Large-scale meta-analyses involving over 13,000 patients have consistently demonstrated that a low pretreatment HALP score is associated with significantly worse overall and disease-related survival across diverse malignancies, including gastrointestinal, thoracic, and genitourinary cancers [17, 18, 20, 21]. Our findings extend these observations specifically to LARC patients treated with contemporary multimodal therapy, a population in which robust prognostic markers are still limited.

From a mechanistic perspective, each component of the HALP score plays a well-established role in cancer progression and treatment response. Low hemoglobin levels reflect cancer-related anemia, contributing to tumor hypoxia, angiogenesis, and resistance to radiotherapy and chemotherapy [22]. Serum albumin serves as a surrogate marker of systemic inflammation and nutritional reserve; hypoalbuminemia is frequently associated with elevated pro-inflammatory cytokines such as IL-6 and tumor necrosis factor alpha (TNF-α), linked to impaired immune competence and poor oncologic outcomes [23]. Lymphocytes are central to antitumor immune surveillance, and lymphopenia indicates weakened cellular immunity, which may compromise tumor control, particularly in the neoadjuvant setting [24, 25]. Conversely, elevated platelet counts reflect a pro-inflammatory and pro-thrombotic state that facilitates tumor growth, angiogenesis, immune evasion, and metastatic dissemination [26]. By mathematically integrating favorable (hemoglobin, albumin, lymphocytes) and unfavorable (platelets) factors, HALP captures the net balance between host defense and tumor-promoting inflammation more effectively than single-ratio indices. Importantly, our comparative analysis demonstrated that HALP outperformed commonly used inflammatory markers, including NLR, PLR, SII, and PIV, in predicting both OS and DFS, as evidenced by higher C-index values, superior time-dependent AUC, and lower Brier scores. This finding aligns with prior colorectal cancer studies indicating that HALP provides more stable prognostic discrimination across different stages and treatment strategies [27]. Moreover, the strong association between low HALP and a globally unfavorable inflammatory profile in our cohort—characterized by higher NLR, PLR, SII, SIRI, and PIV—suggests that HALP may serve as an integrative surrogate of systemic immune–nutritional derangement rather than a redundant marker. Collectively, these data support HALP as a biologically relevant and clinically practical biomarker that complements traditional staging systems and enhances individualized risk stratification in patients with LARC.

**Table 5:** Comparison of the predictive value of the nomogram, TNM system, and clinical model

**Models**	**C-index**			**IDI**			**NRI**	
	**Value**	**95% CI**		**Difference**	***P* value**		**Difference**	***P* value**
OS								
Nomogram	0.705	(0.661, 0.749)		0.072 (0.011, 0.129)	0.003		0.209 (0.127, 0.289)	<0.001
TNM system	0.646	(0.567, 0.725)		--0.052 (--0.114, --0.003)	<0.001		--0.162 (--0.216, --0.104)	<0.001
Clinical model	0.682	(0.632, 0.728)		Ref.			Ref.	
DFS								
Nomogram	0.693	(0.628, 0.745)		0.081 (0.015, 0.132)	0.002		0.228 (0.115, 0.332)	<0.001
TNM	0.616	(0.607, 0.717)		--0.061 (--0.122, --0.003)	<0.001		--0.171 (--0.254, --0.132)	<0.001
Clinical model	0.662	(0.539, 0.693)		Ref.			Ref.	

Abbreviations: C-index: Concordance index; CI: Confidence interval; DFS: Disease-free survival; IDI: Integrated discrimination improvement; NRI: Net reclassification improvement; OS: Overall survival.

Beyond its biological relevance, the most significant clinical implication of this study lies in the robust translational value of the HALP score and its integration into a practical prognostic nomogram for patients with LARC. Consistent with extensive evidence across solid tumors, including colorectal, gastric, lung, breast, and esophageal cancers, our findings confirm that a low pretreatment HALP score is independently associated with inferior survival outcomes, reflecting a state of systemic inflammation, impaired immune competence, and malnutrition [18, 19, 22]. Multiple large-scale meta-analyses have demonstrated that HALP is a powerful and reproducible prognostic indicator across diverse malignancies, often outperforming conventional inflammatory indices such as NLR and PLR [18, 22]. Our results extend these observations to the LARC population treated with nCRT, a setting characterized by significant outcome heterogeneity despite similar TNM stages. Importantly, this study advances beyond biomarker validation by translating HALP into clinically applicable nomograms for predicting OS and DFS. By integrating HALP with key pathological factors, including ypT stage, ypN status, and pretreatment CEA level, the nomogram models demonstrated superior discriminative performance compared with the TNM staging system alone, as evidenced by higher C-index values and significant improvements in integrated discrimination improvement (IDI) and NRI. Similar inflammation-based nomogram approaches have shown enhanced prognostic accuracy in colorectal cancer, nasopharyngeal carcinoma, breast cancer, and digestive system malignancies, highlighting the added value of incorporating host-related systemic factors into traditional tumor-centric models [22, 24, 26, 27]. These findings reinforce the notion that anatomical staging alone is insufficient to fully capture prognosis, particularly in patients undergoing multimodal treatment. From a clinical perspective, HALP-based risk stratification may facilitate individualized postoperative management. Patients with low HALP scores represent a vulnerable subgroup with heightened inflammatory burden and compromised nutritional reserve, who may benefit from intensified surveillance, early nutritional and anti-inflammatory interventions, or tailored adjuvant strategies [23, 28, 29]. Conversely, patients with high HALP scores may achieve favorable outcomes and potentially avoid overtreatment. Notably, HALP relies exclusively on routine blood parameters, ensuring low cost, wide availability, and excellent feasibility across institutions with varying resources. Collectively, our findings support the HALP-based nomogram as a practical and cost-effective tool for precision prognostication in LARC, complementing the TNM system and advancing individualized clinical decision-making.

Postoperative complications remain a significant challenge following colorectal surgery and are closely associated with increased morbidity, prolonged hospital stays, readmissions, sepsis, and mortality, with surgical site infection (SSI) being the most frequent complication in this setting [30]. Growing evidence suggests that host-related factors, particularly systemic inflammation and metabolic reserve, play a critical role in determining postoperative outcomes. In this context, emerging biomarkers such as butyrylcholinesterase (BuChE) have garnered increased attention. BuChE is considered an integrative marker of inflammatory status, nutritional reserve, and hepatic synthetic function, and recent studies have demonstrated that reduced preoperative BuChE levels are significantly associated with higher rates of postoperative complications following colorectal surgery [31]. These findings are conceptually consistent with inflammation-based composite indices, including HALP, which reflect the balance between host immunity, nutrition, and systemic inflammatory burden. Concurrently, advancements in artificial intelligence, particularly deep learning algorithms, have shown promising results in colorectal cancer diagnosis through automated histopathological image classification, enhancing diagnostic accuracy and reproducibility [32, 33]. Although postoperative complications, BuChE levels, and deep learning–based diagnostic tools were not evaluated in the present study, integrating novel enzymatic biomarkers and artificial intelligence–assisted pathology with established inflammatory prognostic models may represent an important future direction for optimizing perioperative risk stratification and personalized management in colorectal cancer patients.

Several limitations of this study should be acknowledged. First, this investigation was retrospective in nature, which inherently introduces potential selection bias and limits the ability to infer causality between the HALP score and survival outcomes. Although we included a relatively large multicenter cohort and applied multivariate analyses to adjust for known confounders, residual and unmeasured confounding cannot be entirely excluded. Second, all participating centers were located in China, and the patient population was ethnically homogeneous. Given that baseline inflammatory and nutritional profiles may vary across regions and populations, the generalizability of our findings to Western or other non-Asian cohorts requires further external validation. Third, the optimal cut-off value for HALP remains a subject of debate. In this study, patients were dichotomized using the median HALP value, a strategy that enhances internal stability but may limit direct comparability with other studies employing ROC-based or outcome-driven thresholds. Establishing standardized, disease-specific cut-off values through large prospective cohorts will be essential for broader clinical implementation. Fourth, HALP was assessed only at a single pretreatment time point. Dynamic changes in inflammatory and nutritional status during nCRT or postoperative recovery may provide additional prognostic information but were not captured in this analysis. Fifth, although HALP demonstrated superior prognostic performance compared to other inflammatory indices, it remains a surrogate marker of systemic immune-nutritional status and does not directly reflect tumor-specific biological mechanisms. Correlative analyses integrating HALP with molecular, immunological, or cytokine profiling were beyond the scope of this study. Sixth, because survival time was measured from surgery and inclusion required the completion of neoadjuvant therapy and resection, the observed associations apply to patients who reached surgery and may not be generalizable to those who did not complete treatment. Finally, while the proposed nomograms showed good discrimination and calibration, they were validated only internally. Prospective studies with external validation, ideally incorporating longitudinal biomarker assessments, are warranted to confirm the clinical utility and robustness of HALP-based prognostic models in patients with LARC.

## Conclusion

In conclusion, this multicenter study demonstrates that the HALP score is a robust and independent prognostic biomarker for patients with LARC treated with nCRT. HALP consistently outperformed other inflammatory indices and, when integrated into nomograms, significantly improved prognostic accuracy beyond TNM staging. Due to its simplicity, low cost, and clinical availability, HALP-based models offer a practical tool for individualized risk stratification and postoperative management in LARC.

**AI writing statement:** No AI writing assistance was utilized in the production of this manuscript.

## Supplemental data

Supplemental data are available at the following link: https://www.bjbms.org/ojs/index.php/bjbms/article/view/13845/4120.

## Data Availability

The data that support the findings of this study are available on reasonable request from the corresponding author.
